# A Dynamic Probabilistic Based Broadcasting Scheme for MANETs

**DOI:** 10.1155/2016/1832026

**Published:** 2016-02-25

**Authors:** Kannan Shanmugam, Karthik Subburathinam, Arunachalam Velayuthampalayam Palanisamy

**Affiliations:** Department of CSE, SNS College of Technology, Coimbatore, Tamil Nadu 641 035, India

## Abstract

MANET is commonly known as Mobile Ad Hoc Network in which cluster of mobile nodes can communicate with each other without having any basic infrastructure. The basic characteristic of MANET is dynamic topology. Due to the dynamic behavior nature, the topology of the network changes very frequently, and this will lead to the failure of the valid route repeatedly. Thus, the process of finding the valid route leads to notable drop in the throughput of the network. To identify a new valid path to the targeted mobile node, available proactive routing protocols use simple broadcasting method known as simple flooding. The simple flooding method broadcasts the RREQ packet from the source to the rest of the nodes in mobile network. But the problem with this method is disproportionate repetitive retransmission of RREQ packet which could result in high contention on the available channel and packet collision due to extreme traffic in the network. A reasonable number of routing algorithms have been suggested for reducing the lethal impact of flooding the RREQ packets. However, most of the algorithms have resulted in considerable amount of complexity and deduce the throughput by depending on special hardware components and maintaining complex information which will be less frequently used. By considering routing complexity with the goal of increasing the throughput of the network, in this paper, we have introduced a new approach called Dynamic Probabilistic Route (DPR) discovery. The Node's Forwarding Probability (NFP) is dynamically calculated by the DPR mobile nodes using Probability Function (PF) which depends on density of local neighbor nodes and the cumulative number of its broadcast covered neighbors.

## 1. Introduction

Designing an efficient routing protocol is one of the most demanding tasks in Mobile Ad Hoc Networks [1, 2]. In MANET, the route is a path which consists of multiple hops made with the help of intermediate nodes available to transmit the mobile data packet from the route initiated source mobile node to the targeted mobile node. The unique characteristics of MANET like mobility, dynamic topology, and resource sharing will make the routing process a challenging one. The mobility of the mobile nodes results in highly dynamic topological network in which the path failures are caused frequently. Due to shared wireless channel, the mobiles nodes are offered with low and variable amount of bandwidth. This helps to perform the communication between the mobile nodes; consequently this will affect the data packet transmission and may lead to considerable amount of loss in throughput. Hence, the routing protocol must be designed to adapt the dynamic changes in topology of network along with the capability to reduce the route request packet transmissions over data packet transmission. Thus, it can increase the available bandwidth to perform the data packet transmission in a much effective way.

As a result of various research works, MANET is possessing variable number of good and effective routing protocols [3, 4] over a decade. By understanding the process of route discovery and routing table update, the routing protocols of MANET have been derived into the following three major types, namely, proactive protocols (also commonly known as table driven protocols), reactive protocols (also commonly known as on-demand protocols), and hybrid routing protocols which is the combination of best practices in reactive and proactive algorithms.

### 1.1. Proactive Mobile Ad-Hoc Network Routing Protocols

As described in [3–6] table driven routing protocols always maintain the updated and accurate information about the valid routes from each participating mobile node to all other mobile nodes of the current network. Also, the process of updating the topological or routing procedures is broadcasted throughout the mobile network in every interval of time for the purpose of maintaining the consistency of MANET. Maintaining the updated routing information from a mobile node to all other remaining mobile nodes of the network will be an added advantage to these protocols. By holding this advantage the Proactive routing protocols can eliminate or reduce the initial delay which is caused while selecting the route for the data transmission. The valid route will be selected immediately from the updated routing table. However, proactive routing protocols have some demerits like producing high amount of control packet traffic to perform regular routing table update process rather than data packet transmission [7, 8]. Destination-Sequence Distance Vector (DSDV) [9] and Optimized Link State Routing (OLSR) [10] are the well-known examples for table driven routing protocols.

### 1.2. Reactive Mobile Ad Hoc Network Routing Protocols

As described in [11, 12] reactive routing protocols establishe the valid path from the source initiated mobile node to targeted mobile node by performing simple flooding of broadcasting the RREQ mobile control packets from the source initiated mobile node to all other remaining nodes of the network when they are needed. Unless there is a need for route to enable the data communication the on-demand routing protocols will not perform route discovery. However, the discovered route has been maintained by following some route maintaining procedures after receiving the acknowledgement (RREP) for route establishments. When compared to proactive routing protocols, the resource utilization by these protocols is very low for routing table update. However, these protocols are taking their own time to find out a new valid path, which leads to long delay before starting the data packet transmission. Ad Hoc On-Demand Distance Vector [11] and Dynamic Source Routing [12] are well-known frequently used protocols of reactive routing protocols.

### 1.3. Hybrid Routing

As defined in [13–16] these routing protocols combines the features of both reactive and on-demand routing strategies. Basically, these kinds of routing protocols split the whole mobile network into group of clusters, also known as zones. Proactive routing strategy is performed inside the zone and it is responsible for the process of identifying the new routes and maintaining all the identified valid routes in intrazone. On the other side reactive routing strategy is performed between the clusters or zones. Every cluster will be managed by one among the mobile nodes of a particular zone known as cluster head.

### 1.4. Broadcasting in MANETs

Broadcasting is the simple and basic process in Mobile Ad Hoc Networks in which the same packet has been transmitted from the sender node to all the remaining nodes of the network. Due to limited radio range of mobile nodes, the MANET is multihop in nature. Hence, the packet which is transmitted from the valid mobile source cannot reach the target in a single hop. So, here some other nodes of the network are required to forward the source transmitted packet to the destination. These nodes are often known as intermediate nodes. In MANET, the process of choosing the intermediate node is the most important factor because these nodes will use the valuable resources of the network like battery power and bandwidth. Also, the process of intermediate node selection will reduce or avoid the redundancy in packet forwarding process.

There are two basic models which are used in broadcasting with respect to physical layer, namely, one-to-many model in which the source transmitted packet will be sent to the other neighbor mobile nodes of the transmission initiated node (neighbor nodes are the mobile nodes which come under the radio range of the source node) and one-to-one model in which the source transmitted packet will be given to a specific neighbor only. As discussed in [17, 18] the broadcasting process has many advantages with respect to the network layer. However, the broadcasting mechanism in Mobile Ad Hoc Network acts as a backbone of several protocols which are available in the network layer. The broadcasting serves several purposes like paging of a node, packet forwarding to the entire mobile network, network management, overhead control, route discovery, and maintenance. A reasonable number of broadcasting strategies are supported by several authors [19, 20] which includes probability based routing, counter value based routing, location based routing, and neighbor knowledge based routing. In probabilistic based routing approach the intermediate nodes forward the packet to its neighbor based on some fixed probability value.

In this work, we have introduced a probabilistic based routing method which is commonly known as dynamic probabilistic route discovery. Here, the mobile nodes can calculate their forwarding probability for respective data packet in a dynamic manner and this calculation is performed by using probability function which depends on density of the local neighbor and the cumulative number of its neighbors.

## 2. A Dynamic Probabilistic Route Discovery Scheme

The task of including the neighbor information gathering algorithm to enable the functionalities of DPR scheme is a difficult one because the probability function (PF) of the DPR approach depends on the density and number of neighbor nodes of the corresponding node. The DPR approach first divides the network into two groups based on the density using local density of the corresponding node and which are known as sparse and dense networks. The mobile elements which are located in the sparse area are able to rebroadcast the source broadcasted packet with the forwarding probability FP = 1 whereas the mobile nodes in the dense region will be permitted to rebroadcast the broadcasted packet with the forwarding probability of FP < 1. In [2, 18, 21, 22] the broadcasting approaches are proposed with forwarding probability to coordinate the forwarding process of the broadcasted packet which is calculated based on the local density and the number of local neighbors.

With respect to the discussion made in [22, 23] for the design and implementation of self- pruning approach, every node in the network (e.g., node *S*) piggybacks the set of its neighbors nodes *N*(*S*), before forwarding the broadcasted packet. The number of neighbor nodes of a corresponding node is calculated as 1-number of hops. When the other node *R* in the network receives the broadcasted packet from the mobile node *S* for the first time, it decides to rebroadcast the packet to its neighbor, based on the result of the set *N*(*R*) − *N*(*S*). Here, *N*(*R*) denotes the set of neighbor nodes of the node *R*. If the resultant set of *N*(*R*) − *N*(*S*) is empty (i.e., when node *R* is also having the same neighbor as node *S*), then the node *R* desists from retransmitting the broadcast packet to its neighbor node.

In [21, 24], the authors proved that the network overhead with respect to data packet communication is high if the network uses 1-hop neighborhood information, especially in dense region of the mobile network. Hence, the authors suggested that each mobile node must have 2-hop neighborhood information for the purpose of reducing the quantity of mobile nodes involved in the process of forwarding the mobile data packet which could be attained by periodically exchanging the control packets among the neighbor nodes.

The developed efficient route discovery strategy will be useful for finding the shortest route to the targeted mobile node by avoiding the need of GPS receivers and global topological information while revealing aggressive system concert (e.g., throughput) with least routing overhead, collision rate, and end-to-end delay.

**Figure 1:**
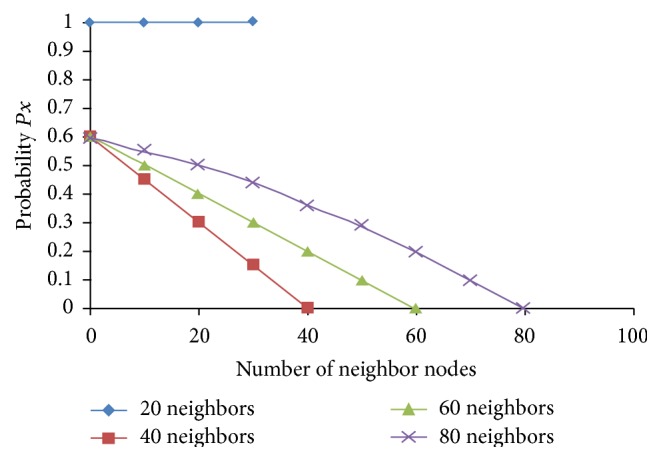
FP at node *N* versus number of covered neighbors.

For the purpose of analyzing the performance of the proposed approach DPR, the traditional DSDV routing algorithm with neighborhood information gathering approach has been used. The information gathering approach uses the periodic control packet transmission among the neighbor nodes to collect the 2-hop neighbor information at a corresponding node. The average neighbor density to be decided as 25 (*n*_*f*_ = 25) for distinguishing the dense and sparse region in the mobile network. Hence, in the DPR scheme, with respect to the equation shown in (1), a node in the dense region (where number of neighbor is greater than 25, i.e., *N* > *n*_*f*_) could rebroadcast the broadcasted packet with the forwarding probability FP < 1 whereas the node could be able to rebroadcast the packet with forwarding probability FP = 1, when it is in sparse network (where number of neighbor is less than 25, i.e., *N* < *n*_*f*_).  Figure 1 shows the packet forwarding at four different nodes in typical dense network along with different number of neighbor sets.

### 2.1. The Forwarding Probability in DPR

Let us consider the total number of neighbor mobile nodes at a node *X* as *n* and let the total number of neighbor mobile nodes of *X* that are located inside the radio range of *X* and which are covered by the broadcast as *n*_*c*_. Then the forwarding probability at the mobile node *X* is

(1) $$\begin{array}{c} P_{x}=\left\{\begin{array}{cc} 1; & n\leq n_{f},\\ 1-e^{-}\left(\frac{n-n_{c}}{n}\right); & n>n_{f}. \end{array}\right. \end{array}$$

## 3. Experimentation and Discussions

The performance evaluation and the result analysis are obtained by the DPR approach, with the implementation of AODV routing protocol in NS2 simulator [25] which has been modified to enhance the process of DPR approach. The simulation has been performed on DPR with respect to the varying density of the network and offered load at different levels. Performance metrics which are considered for this analysis are network overhead during the routing process, rate of collision which will be calculated during the packet transmission, network throughput which will be obtained by achieving the successful delivery of mobile data packet, and the end-to-end delay which will increase the delay in the successful packet delivery.

The following assumptions, which have been widely adapted, are used throughout this work and the simulation parameters are listed in Table 1.

- Each and every mobile node is considered to possess enough energy to function throughout the simulation time.
- Throughout the simulation time the number of nodes is fixed for the assumed topology.
- Transmissions may interfere with each other; however, any node can always successfully decode the transmission provided.
- All mobile nodes are considered as homogeneous nodes.
- All mobile nodes participate fully in the routing process of the network.
- Any mobile node in the network can initiate the routing process which has a data packet to be transmitted.

In this simulation, some threshold values are considered and used to perform the comparison of performance between the existing and proposed algorithms. The threshold values are chosen based upon the study performed in variety of existing results. These values are commonly used by many researchers.

### 3.1. Impact of Network Density

This part of simulation analyzes the effect of the network density which will be dynamically changing during the simulation process. The density of the mobile network is differed by increasing and decreasing the number of participating mobile nodes which are deployed over a 1250 m × 1250 m area for each simulation. The mobility of the mobile nodes is randomly fixed from 0 to 25 m/s. for every simulation test. The number of valid communication links between the source and destination node has been considered and those links are randomly selected by the corresponding algorithm.

#### 3.1.1. Overhead on Routing

**Figure 2:**
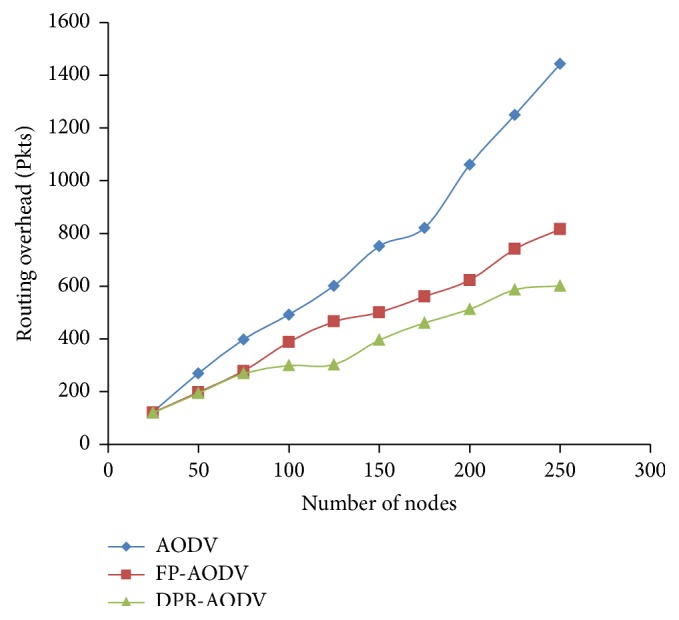
Overhead on routing versus number of mobile nodes.

**Figure 3:**
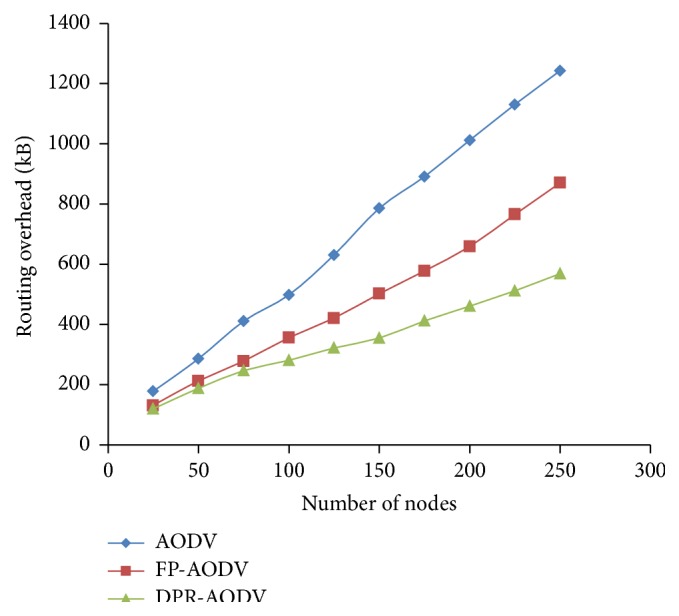
Overhead in bytes versus number of mobile nodes.

The performance of the three routing protocols, namely, AODV, FP-AODV, and DPR-AODV in terms of overhead occurred during the routing in the network with respect to density of the mobile network is demonstrated in Figure 2. The overhead in all the three routing protocols is linearly increased when the density of the network increases. However, DPR outperforms AODV and FP because of the fact that in DPR the forwarding probability (FP) is decided based on the local density of the mobile node and number of neighbor nodes, which causes redundancy in the number of control packet transmission and leads to the overall overhead reduction. The piggybacked list of 2-hop neighbor information has been forwarded along with RREQ packets in DPR; as a result the overhead is amplified with respect to number of bytes of data transmitted. By considering this case, Figure 3 illustrates the operation of the considered routing protocols with respect to overhead in terms of bytes. Even though the DPR produces less overhead when compared to the remaining two protocols with respect to number of packets transmitted as revealed in Figure 2, the decrease in overhead of DPR is comparatively low.

#### 3.1.2. Collision Rate

**Figure 4:**
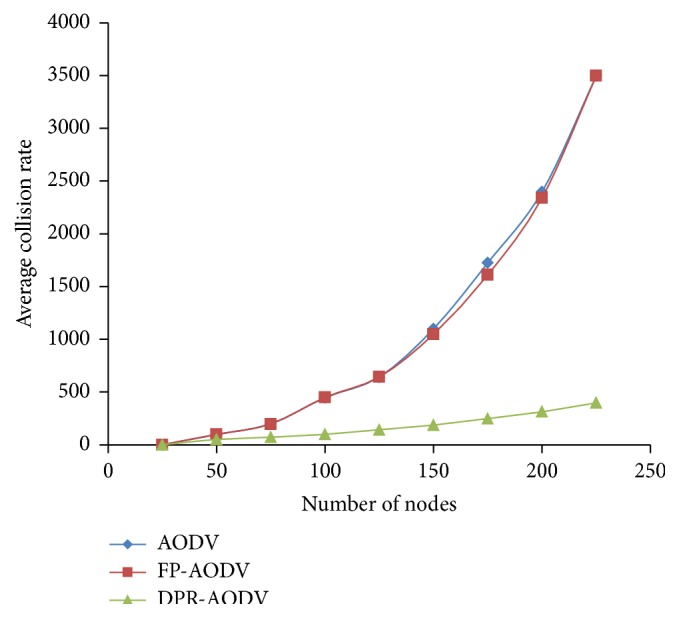
Average collision rate versus number of mobile nodes.

In MANET, increasing in packet transmission also increases the traffic and congestion which leads to the packet collision.  Figure 4 shows the average collision rate of all the three routing protocols with respect to network density. Since the communication medium is shared by both control and data packet transmission, the probability of the collision also increases without having proper approach to control the retransmission of the route request packet. The figure shows that the proposed approach DPR outperforms DSDV and FP-DSDV.

#### 3.1.3. Throughput and Connectivity

**Figure 5:**
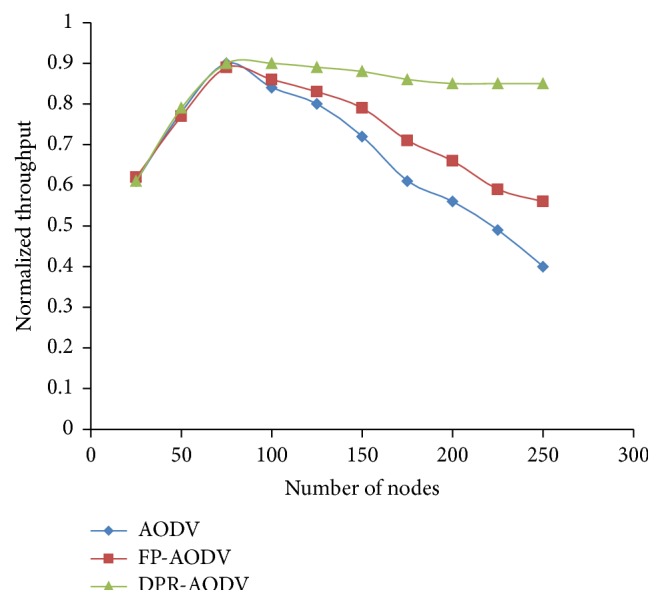
Normalized throughput versus number of mobile nodes.

**Figure 6:**
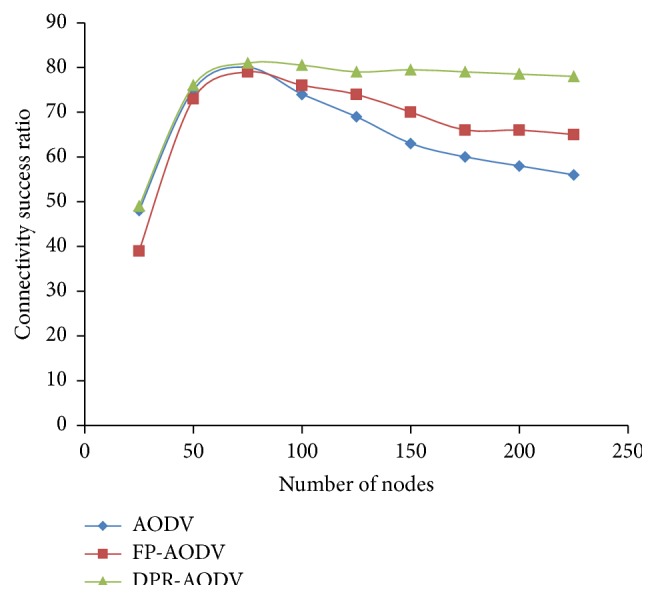
Success ratio on connectivity versus number of mobile nodes.

Normalized throughput of the network which is plotted against the density of the network is shown in Figure 5. The figure proves that the throughput of the network is very low when the region of the network is sparse. The reason behind this is poor connectivity ratio among the mobile nodes of the sparse region (Figure 6). On the other hand, the connectivity ratio is better, when the network becomes dense. This increases the number of control packet (RREQ) retransmission and leads to insufficient bandwidth for data packet transmission. Hence, there is a sudden drop of the network throughput even in the dense network. Therefore, if there are any measures taken towards controlling the repeated retransmission of route request packet, then the degradation of the throughput could be reduced. However, Figures 4 and 6 show that the performance of the DPR is relatively good even in dense network, when compared with DSDV and FP-DSDV. Also, it reveals that the DPR effectively reduces the retransmission of route request packets (RREQ).

#### 3.1.4. End-to-End Delay

**Figure 7:**
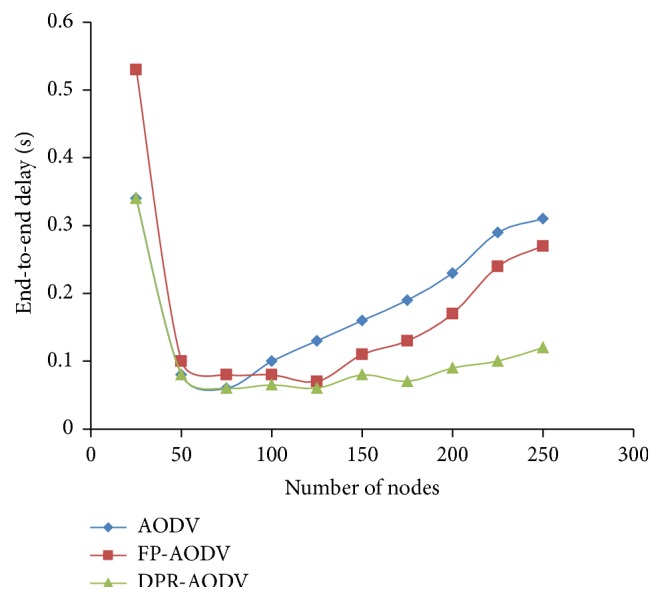
End-to-end delay versus number of mobile nodes.

Figure 7 gives an idea about the end-to-end delay in the dense and sparse area of the available mobile network. Also, it portrays that the delay is relatively high for all the three protocols in both sparse and dense regions of the network. This is because in sparse region the connectivity ratio is less. Due to this, the failure of RREQ packets to reach the destination is high. Subsequently, the end-to-end delay is also increased for every mobile node whereas in dense network, due to high contention which is rooted by massive unnecessary retransmission of control packets, the destination originated packets fail to reach the destination. This leads to increase in delay. However, the performance of the DPR is comparatively well against AODV in sparse network and performs greatly against FP-AODV whereas the performance of DPR is far better than all other protocols when the network is dense.

To analyze the performance of the mobile node with respect to the offered load of all the three routing protocols, here we have considered different number of source-to-destination connections known as flows. The load on the mobile network has been assorted like 1,5, 10,15,…, 60 flows.

### 3.2. Impact of the Load Offered to the Mobile Network

#### 3.2.1. Overhead

**Figure 8:**
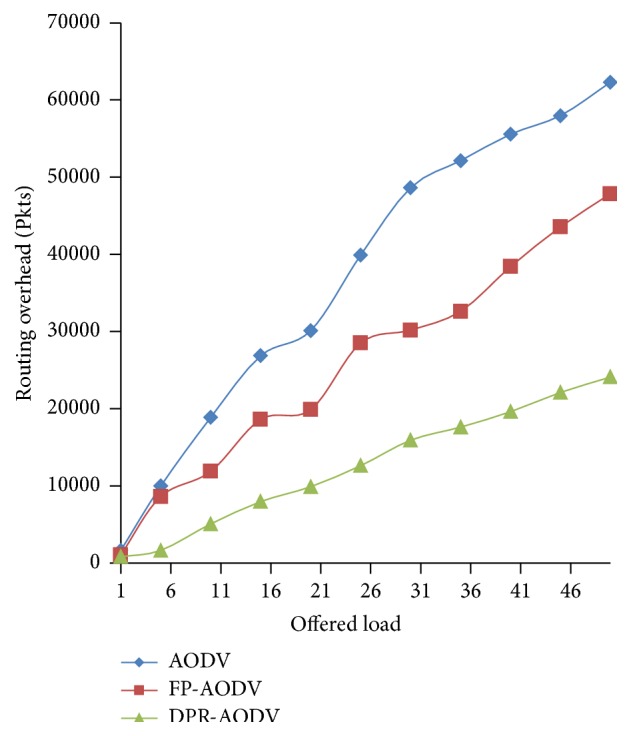
Overhead versus offered Load (in terms of number of packets).

**Figure 9:**
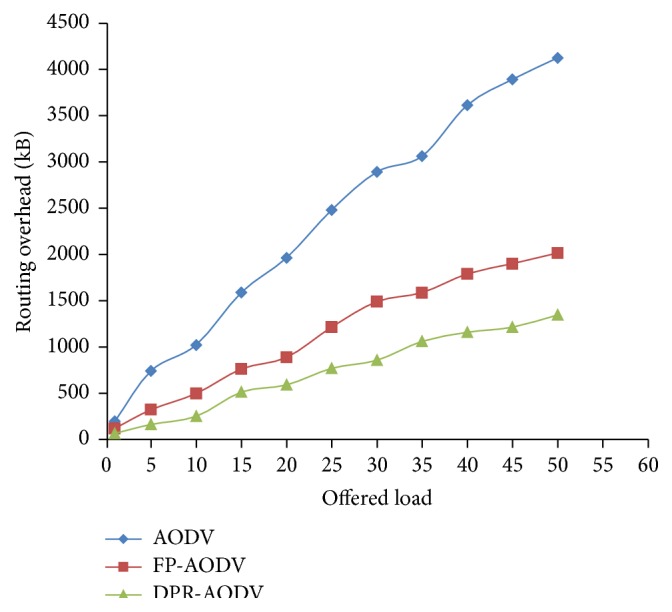
Overhead versus offered load (in terms of number of transmitted bytes).

Figures 8 and 9 show that the overhead produced during the routing by all the three protocols which are compared against the dynamic loads varies among the different mobile networks. Figures 8 and 9 show that the overhead is increased relatively in all the three protocols. However, the proposed approach DPR performs well against all the other protocols in terms of both packet transmission and byte transmission. The proposed approach outfits the route discovery operation only to smaller amount of mobile nodes which are contributing in the RREQ forwarding.

#### 3.2.2. Collision Rate

**Figure 10:**
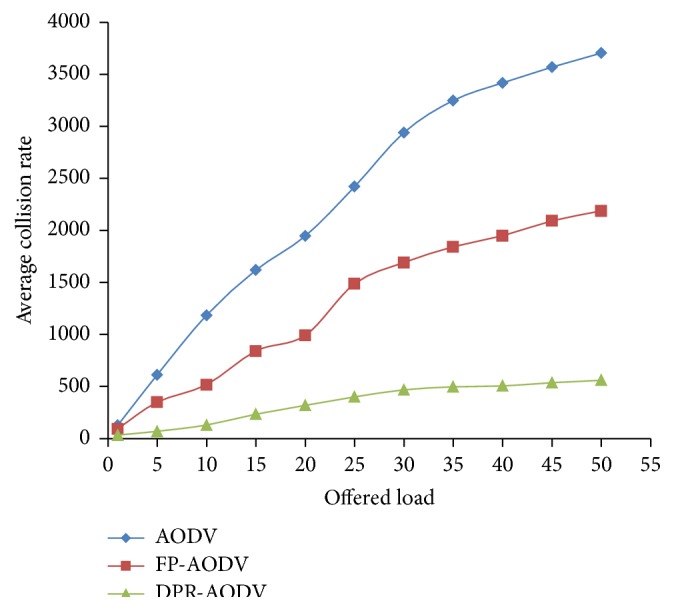
Average collision rate versus offered load.

As the overhead represented in the above section, collision rate also linearly increases for all the routing protocols when the dynamically given load has been increased as depicted in Figure 10. The reason behind this is the number of flows has been amplified when increasing the load, which leads to generation and retransmission of route request packets. Hence, the collision rate automatically increases. However, it is noticed from Figure 10 that the DPR performed well when compared with DSDV and FP-DSDV. This is because the DPR drops the good number of route request packets which is forwarded by the forwarding mobile node which is based on the forwarding probability. The FP is calculated based on the home density and the total number of contributing local neighbors. Hence, the channel contention is reduced which leads to less collision rate in DPR.

#### 3.2.3. Normalized Throughput

**Figure 11:**
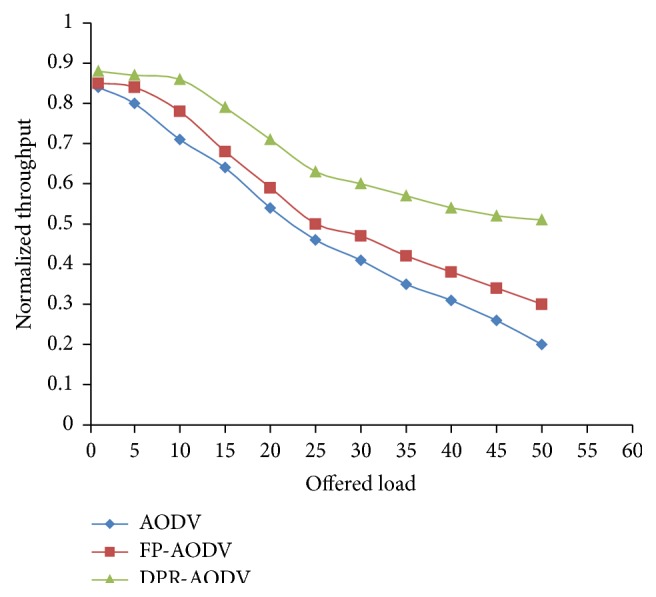
Normalized network throughput versus offered load.

**Figure 12:**
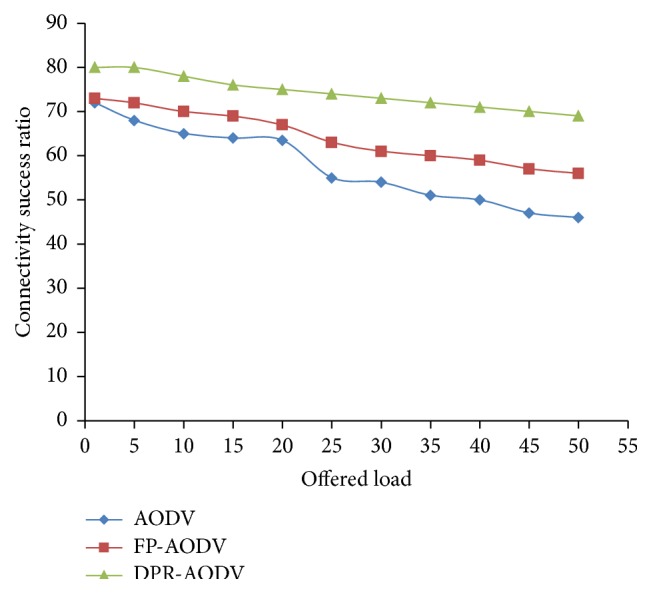
Connectivity success ratio versus offered load.

In Figure 11, we have demonstrated the throughput of the network with respect to offered load. Here the offered load has been increased by growing the number of participating flows from 1 to 60. The protocol represented in the figure clearly discloses that the success rate of the network is high when the offered load is squat. When we increase the offered load then the throughput also decreases, respectively, and it is reduced irrespective of routing algorithms. This is because when we increase the load, it increases the number of mobile nodes which will initiate the route discovery process. As a result, the generation and transmission of route request packet also increases and it leads to high contention in the data communication channel and packet collision. In Figure 12, the connectivity success ratio is depicted and it reveals that the DPR outperforms when compared with all other routing protocols. As a result of high connectivity ratio with respect to result shown in Figure 12, the DPR produces high throughput when compared with other protocols as depicted in Figure 11.

#### 3.2.4. End-to-End Delay

**Figure 13:**
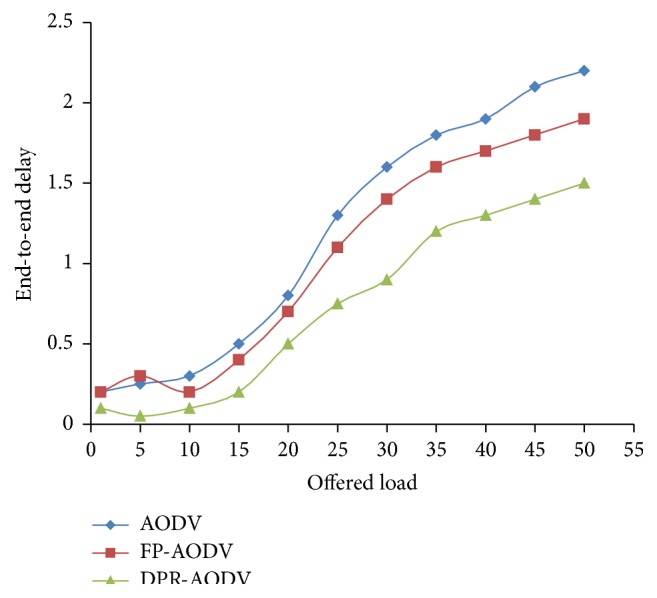
Delay versus offered load.

As shown in Figure 13, the delay increases when the dynamic load increases. The reason is when the load of the network is enlarged it will increase the contention on the network. Hence, the route failure occurs frequently, when the contention is high. However, the DPR performs relatively well when compared with other routing protocols.

## 4. Conclusions

The proposed schemes open a novel approach for probabilistic based broadcasting approach for route discovery known as dynamic probabilistic based routing (DPR). In DPR, the packets are forwarded to the neighbor node with dynamically computed probability called as forwarding probability (FP). The probability function is calculated based on the density of the local neighbors and cumulative amount of neighbor mobile nodes. Hence, it is vital to identify the dense and sparse regions. The performance of the DPR has been compared against two more routing protocols traditional DSDV and FP-DSDV. The performance of the various routing protocols has been compared using various quality of service parameters of MANET, namely, overhead, average collision rate, end-to-end delay, and network throughput. The system parameters like impact of network density and impact of offered load are considered for assessing the performance of the planned routing approach.

The simulation results for impact of the varying network density for all the three considered routing methods with respect to the overhead and collision rate degrade the performance when the density is increased. However, the order of performance of all three routing approaches is DPR, FP-DPR, and DSDV for collision overhead and overhead with respect to network density. Here, these performance measurements are analyzed by considering the packet transmission in terms of both packet and bytes. When considering end-to-end delay and network throughput, again the DPR outperforms DSDV and FP-DSDV. In another simulation for impact of offered load the results prove that the overhead and collision rates are increased when increasing the offered loads in the mobile network. However, the proposed approach DPR shows the improved performance compared to the other two routing protocols. The same level of performance is achieved by DPR for the case of network throughput and end-to-end delay.

## Figures and Tables

**Table 1:** Simulation parameters.

Parameter	Value
MAC protocol	802.11
Packet type	UDP
Traffic type	CBR
Number of mobile nodes	250
Topography area	1000 × 1000 M
Protocol	DSDV
Mobility model	Random waypoint
